# Synthesis of a HDAC inhibitor–nanogold probe for cryo-EM visualization in class I HDAC co-repressor complexes

**DOI:** 10.3762/bjoc.22.35

**Published:** 2026-03-17

**Authors:** Wiktoria A Pytel, John W R Schwabe, James T Hodgkinson

**Affiliations:** 1 Institute for Structural and Chemical Biology, University of Leicester, Leicester LE1 7RH, UK; 2 Department of Molecular and Cell Biology, University of Leicester, Leicester LE1 7RH, UK; 3 School of Chemistry, University of Leicester, Leicester LE1 7RH, UK

**Keywords:** CI-994, co-repressor complex, CoREST, cryo-EM, gold nanoparticle, HDAC

## Abstract

Class I histone deacetylases (HDACs 1–3) serve as catalytic subunits within seven multiprotein co-repressor complexes, each of which has distinct functions in the cell. We report the synthesis of a HDAC inhibitor–nanogold probe, derived from the class I HDAC inhibitor CI-994, for cryo-electron microscopy (cryo-EM) visualization of the HDAC catalytic domain within class I HDAC co-repressor complexes. The nanogold probe retained HDAC inhibitory activity comparable to CI-994 against the HDAC1-LSD1-CoREST complex in vitro. In cryo-EM studies, 2D class averages revealed the bi-lobed architecture of the CoREST complex and partial localization of the gold nanoparticle probe to the CoREST complex. However, the probe was not observed in classes showing the side-view of the CoREST complex, limiting unambiguous identification and positioning of the HDAC catalytic domain within the CoREST complex.

## Introduction

Histone deacetylase (HDAC) enzymes catalyze the hydrolysis of acetyl groups from *N*-acetylated lysine residues in histone proteins. HDACs are also capable of the deacetylation of non-histone proteins [1], and the hydrolysis of other acyl functional groups [2]. The human genome encodes 18 histone deacetylases (HDACs), which are divided into two main groups based on their catalytic mechanisms [3]. Eleven HDACs are zinc-dependent enzymes, while the remaining seven, known as sirtuins (SIRT1–7), require nicotinamide adenine dinucleotide (NAD^+^) as a cofactor [3]. HDACs are further classified into four classes: class I (HDAC1–3 and HDAC8), class IIa (HDAC4, 5, 7, and 9), class IIb (HDAC6 and 10), class IV (HDAC11), and the NAD^+^-dependent sirtuins are grouped separately as class III [3].

HDAC1, HDAC2, and HDAC3 of the class I HDACs exist in multiprotein co-repressor complexes in vivo [4]. HDAC1 and HDAC2 exist interchangeably in the CoREST, MIDAC, SIN3, NuRD, MIER, and RERE complexes, while HDAC3 exists in the SMRT/NCoR complex [4]. The protein complex partners govern the nucleosomal substrate specificity [5–6], and each complex has distinct cellular functions [7].

Cryo-electron microscopy (cryo-EM) has revolutionized structural biology by enabling high-resolution, three-dimensional visualization of macromolecular multiprotein complexes in differing functional states. Examples of structure elucidation utilizing cryo-EM for class I HDAC complexes include the MiDAC and SIN3 complexes [8–9]. However, despite these advances, obtaining high-resolution structures of flexible multiprotein complexes can still prove challenging. One such example of this includes the tripartite CoREST complex that encompasses HDAC1/2, the co-repressor of REST (CoREST) and the lysine-specific demethylase 1 (LSD1). Cryo-EM and small angle X-ray scattering revealed that the CoREST complex exists as a bi-lobed structure [10]. Enzyme kinetics studies showed that HDAC1 and LSD1 do not act independently and that their activities and modulation by inhibitors and activators are closely coupled. Both enzymes exist in at least two distinguishable states that differ in their kinetic properties, consistent with the two distinct structural states of the complex observed in the cryo-EM maps [10]. However, the intrinsic flexibility of the CoREST complex has limited the achievable resolution in cryo-EM reconstructions, making it difficult to confidently assign the orientation of the HDAC catalytic domain relative to LSD1 [10]. Understanding the spatial positioning of the HDAC active site within the complex is critical, as it may significantly influence how CoREST engages with nucleosomal substrates.

Nanogold particles (1–5 nm in diameter) are highly electron-dense and provide high contrast in microscopy. They have been successfully applied in immunogold electron microscopy to study protein localization within a cell, as well as in cellular cryo-electron tomography [11–12]. More recently, gold nanoparticles have been used as labeling tools in vitrified cells to locate cellular compartments in cellular cryo-EM [13]. Given the considerable utility of nanogold particles in microscopy, we aimed to synthesize a nanogold-conjugated HDAC inhibitor and evaluate its applicability in single-particle cryo-EM to unambiguously determine the positioning and orientation of the HDAC active site within the CoREST complex.

## Results and Discussion

### Design and synthesis of a HDAC inhibitor–nanogold probe

**Figure 1:**
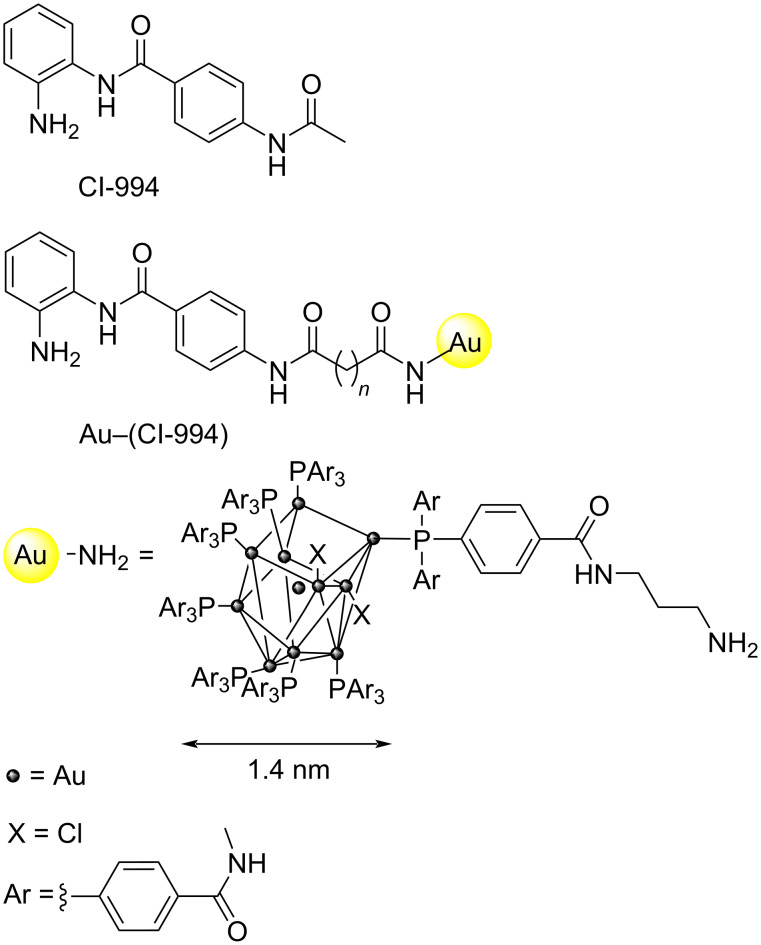
HDAC1–3 inhibitor CI-994; HDAC inhibitor–nanogold probe Au–(CI-994); structure of amine-functionalized 1.4 nm nanogold particle (Au–NH_2_).

For the basis of the HDAC inhibitor–nanogold probe we utilized the class I HDAC inhibitor CI-994 (Figure 1). CI-994 is an inhibitor of HDAC1–3 and it inhibits HDAC1-CoREST-LSD1, HDAC2-CoREST-LSD1, and HDAC3-SMRT complex with IC_50_ values of 0.53 µM, 0.62 µM, and 0.14 µM, respectively [14–15]. Additionally, CI-994, like other benzamide HDAC inhibitors, exhibits slow on/off binding kinetics, hence once bound to the HDAC within the complex it should not readily dissociate [16]. A crystal structure of HDAC2 bound to an analogue of CI-994 (PDB: 4LY1) revealed that the acetamide moiety is oriented outside the HDAC active site [17]. Hence, we decided to functionalize this position with an alkyl linker consisting of 9 carbon atoms to mitigate any steric clashes between the HDAC inhibitor and the nanogold particle, which could be detrimental to the probe binding affinity (Figure 1). Further to this, we previously functionalized this position with linkers for the development of HDAC1–3 proteolysis targeting chimeras (PROTACs) [14,18]. Alkyl-linker lengths of approximately 12 atoms and greater were the most effective degraders [18]. We chose the commercially available amine functionalized nanogold particles (Au–NH_2_, specifically Monoamino-Nanogold^®^ 1.4 nm purchased from Nanoprobes) for our probe design. Au–NH_2_ consists of a gold cluster of eleven gold atoms coordinated by trisarylphosphine ligands with a diameter of 1.4 nm. One of the trisarylphosphine ligands contains the 3-aminopropylamido group, allowing for stoichiometric conjugation with suitable substrates. The small size of Au–NH_2_ minimizes steric hindrance and allows for enhanced spatial resolution, relative to colloidal nanogold particles, thereby facilitating high precision labeling of the HDAC active site.

**Scheme 1:**
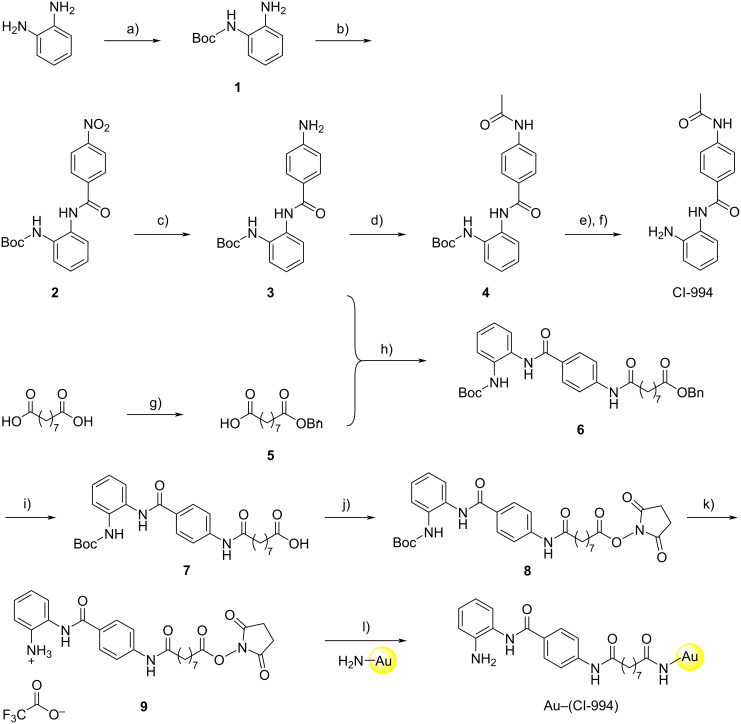
Synthesis of CI-994 and the Au–(CI-994) conjugate. Conditions: a) Boc_2_O, NEt_3_, THF, 0 °C to rt, 19 h, 70%; b) 4-nitrobenzoyl chloride, DIPEA, DCM, 0 °C to rt, 16 h, 74%; (c) H_2_, 10% Pd/C, MeOH/THF 1:1, rt, 17 h, 95%; d) AcCl, NEt_3_, THF, 0 °C to rt, 21 h, 67%; e) TFA, DCM, 0 °C to rt, 20 h; (f) MP-carbonate resin, MeOH, rt, 3 h, 95%; g) benzyl bromide, 1,4-dioxane/DMF 1:1, 90 °C, 19 h, 45%; h) **3**, **5**, HATU, DIPEA, DMF, 0 °C to rt, 40 h, 59%; i) H_2_, 10% Pd/C, THF, rt, 19 h, 99%; j) *N*-hydroxysuccinimide, EDC, DMF, 0 °C to rt, 16 h, 90%; k) TFA, DCM, 0 °C to rt, 18 h, 92%; l) Au–NH_2_, DMSO, rt, 20 h.

CI-994 was synthesized using previously established routes (Scheme 1) [14–15,18]. Intermediates **5**–**7** were prepared in a manner analogous to Smalley et al. [14]. The first step in the linker synthesis for Au–(CI-994) involved a monoprotection of nonanedioic acid with a benzyl group to give **5** which proceeded in moderate yield due to the formation of the dibenzylated by-product. Compound **5** was then coupled to the CI-994 intermediate **3** via HATU-mediated amide bond formation to produce **6** in good yield. Removal of the benzyl protecting group was performed by catalytic hydrogenation and acid **7** was obtained in near quantitative yield. Intermediate **7** was converted to its corresponding NHS ester **8** and residual starting material was removed by column chromatography. The Boc-protecting group was removed under anhydrous conditions in good yield and the structure of **9** was confirmed by NMR spectroscopy. Compound **9** was maintained as the TFA salt for the final step, and was stable for days stored at −20 °C (stability determined by ^1^H NMR, Figures S1–S4 in Supporting Information File 1).

For the conjugation of **9** to Au–NH_2_, an excess of **9** was used to drive the conjugation reaction to completion. Unreacted **9** was then removed by repeated washing with water and buffer, followed by centrifugation (see Supporting Information File 1 for full details). The resulting Au–(CI-994) conjugate was concentrated to a final volume of 1 mL. The concentration of Au–(CI-994) was determined by UV–vis spectroscopy, using absorbance at 420 nm – a characteristic wavelength for nanogold [19]. Based on the Beer–Lambert law, the concentration was calculated to be 2.69 μM. Attempts at further concentration led to precipitation, indicating limited solubility at higher concentrations.

### The HDAC inhibitor–nanogold probe inhibits HDAC enzymatic activity in the CoREST complex

**Figure 2:**
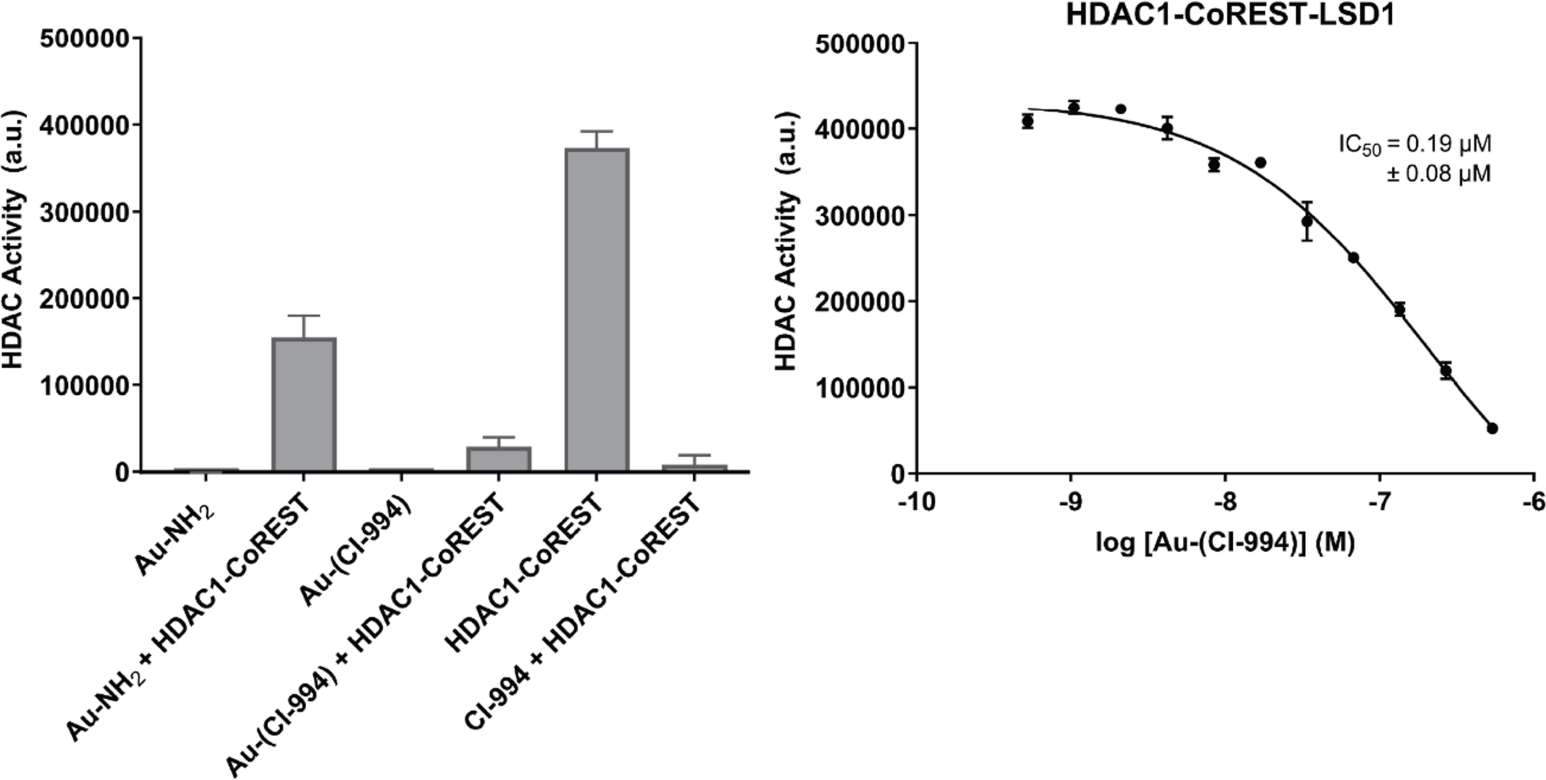
Left: Single point concentration HDAC assay. Concentration of Au–NH_2_ and Au–(CI-994) = 0.54 µM. Concentration of CI-994 = 20 µM. Right: Dose response inhibition of HDAC activity in the CoREST complex with Au–(CI-994). Boc-(Ac)Lys-AMC substrate = 100 µM; CoREST complex = 12.5 nM. Error bars represent ± S.E.M. (*n* = 3). For full assay conditions see Supporting Information File 1.

We first wanted to confirm that the conjugation of CI-994 to the nanogold particle did not significantly affect HDAC inhibition. The HDAC1-LSD1-CoREST complex, incorporating a FLAG tag in CoREST, was expressed and purified from HEK293F cells as previously reported [10]. Fluorescent deacetylase assays were carried out using Boc-Lys-(Ac)-AMC as the HDAC substrate [15,20], which on cleavage by HDAC releases fluorescent 7-amino-4-methylcoumarin. Au–(CI-994) and several controls were evaluated under the HDAC assay conditions in the presence and absence of the CoREST complex (Figure 2).

In the absence of the CoREST complex Au–NH_2_ and Au–(CI-994) did not affect fluorescence under the HDAC assay conditions, implying that the gold nanoparticle does not interfere with the HDAC assay conditions. As expected, 20 µM of CI-994 completely inhibited HDAC activity of the CoREST complex. Notably, Au–(CI-994) also exhibited near-complete inhibition of the HDAC activity in the CoREST complex, even at 0.54 μM. Surprisingly, Au–NH_2_ was found to reduce the HDAC activity of the CoREST complex by nearly 50%. One plausible explanation for this effect is a direct interaction between the gold nanoparticles and solvent-accessible cysteine residues. Cysteines possess thiol side chains that exhibit strong affinity for gold, enabling them to form stable bonds with metal surfaces [21]. Such interactions may disrupt the native conformation of cysteine-containing proteins or peptides, potentially impairing the structural integrity of the CoREST complex and diminishing its deacetylase function. However, the maximal HDAC inhibition by Au–NH_2_ was considerably less compared to Au–(CI-994) and CI-994, suggesting Au–(CI-994), is inhibiting HDAC enzymatic activity by direct competition for the HDAC active site. We next determined the IC_50_ of Au–(CI-994) against the CoREST complex. Au–(CI-994) exhibited an IC_50_ value of 0.19 ± 0.08 µM, this is directly comparable to the IC_50_ value for CI-994, IC_50_ = 0.53 µM for the CoREST complex [14–15]. We noted HDAC activity did not reach 0% with Au–(CI-994) which we speculate was due to precipitation of Au–(CI-994) at higher concentrations. The Au–(CI-994) probe was further characterized using electron microscopy (Figure S5 in Supporting Information File 1). The electron-dense gold nanoparticles appeared prominently in the micrographs due to their intense black contrast. The particles exhibited uniform size distribution, were evenly dispersed across the grid, and showed no signs of aggregation. These studies confirmed the suitability of Au–(CI-994) probe for structural studies with the CoREST complex.

### The HDAC inhibitor–nanogold probe localizes with the CoREST complex in cryo-EM

**Figure 3:**
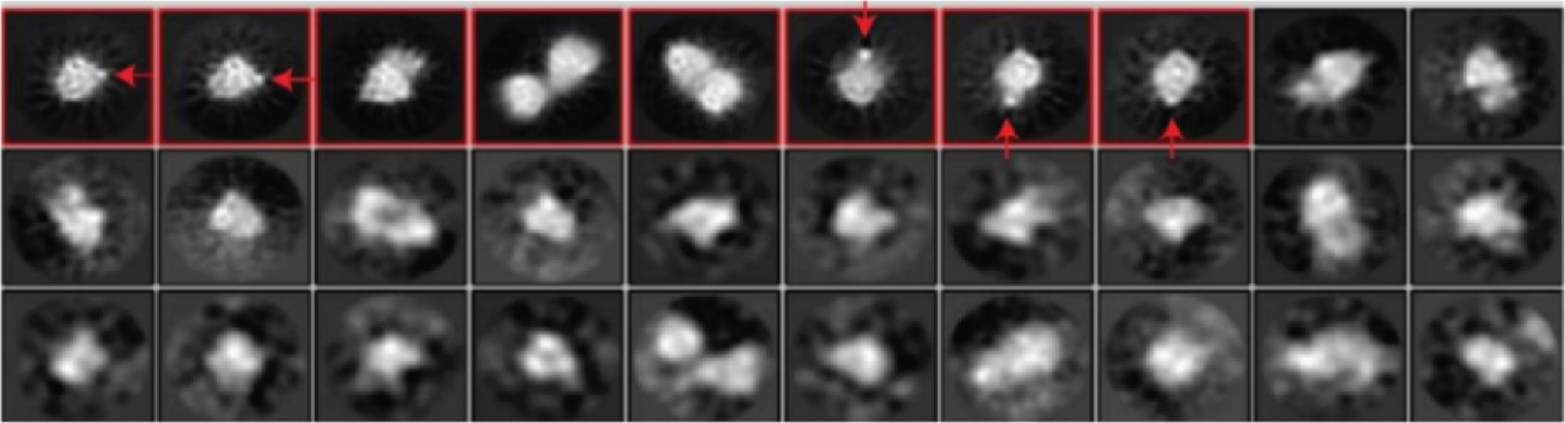
Final round of 2D classification of the cross-linked CoREST complex in the presence of Au–(CI-994) (87,649 particles). Red arrows indicate the position of the Au–(CI-994) probe localized with the CoREST complex. The probe was absent in side-view classes.

To stabilize the HDAC1-LSD1-CoREST complex on the EM grid, glutaraldehyde cross-linking was performed, with successful cross-linking confirmed by SDS-PAGE. The cross-linked ternary complex was incubated with the Au–(CI-994) probe for 2 hours in 2:1 molar ratio to minimize non-specific binding. Screening and data collection were carried out on a Titan Krios microscope. A range of protein particles resembling the CoREST ternary complex were observed, with several displaying a distinct “black dot” indicative of the electron-dense gold nanoparticle (Figure S6 in Supporting Information File 1). However, as expected, Au–(CI-994) was not observed in all protein particles, indicating that the CoREST complex was not fully saturated with the probe. Automated particle picking using TOPAZ identified 370,670 particles from 1,965 micrographs, averaging 189 particles per micrograph (Figure S7 in Supporting Information File 1). Both Au–(CI-994)-bound and unbound particles were picked and subsequently separated during data analysis. Particles were extracted and subjected to multiple rounds of 2D classification to remove artefacts and junk particles. The final 2D classes (Figure 3) revealed a bi-lobed architecture consistent with the published CoREST ternary complex structure [10]. The Au–(CI-994) probe was visible in some classes due to its distinct, brighter contrast. While various orientations of the complex were captured, the probe was absent in side-view classes. These views are especially important for visualizing the active sites of HDAC1 and LSD1, and thus the spatial relationship of the Au–(CI-994) probe to the LSD1 active site. Hence, although the probe localized to the CoREST complex, unfortunately, the positioning and spatial orientation of the HDAC catalytic domain could not be unambiguously determined.

## Conclusion

In summary, we report the synthesis and validation of Au–(CI-994) as a nanogold-conjugated HDAC inhibitor for cryo-EM visualization in class I HDAC co-repressor complexes. Au–(CI-994) effectively inhibits HDAC activity within the CoREST complex in vitro, showing comparable potency to CI-994. The probe was clearly visualized in association with the CoREST complex by cryo-EM; however, its absence in side-view 2D classes prevented precise localization of the HDAC catalytic domain. We speculate that the flexibility of the 9-carbon alkyl linker, and additional flexible linker to the nanogold particles, may have resulted in signal averaging, obscuring the probe’s exact position. Furthermore, although probe localization was evident, complete saturation of the CoREST complex with the probe was not achieved. Future studies will focus on rigidifying the linker and enhancing HDAC binding affinity. Nonetheless, nanogold-labeled HDAC inhibitors could serve as effective fiducial markers in cryo-EM, facilitating the localization of distinct subunits or binding sites within large and flexible multiprotein complexes.

## Supporting Information

File 1 Chemical protocols and characterization data for compounds, biological protocols including cryo-EM grid prep, data collection, and images of micrographs.

## Data Availability

All data that supports the findings of this study is available in the published article and/or the supporting information of this article.
