# Supplementary material for: Synthesis of a HDAC inhibitor–nanogold probe for cryo-EM visualization in class I HDAC co-repressor complexes
Source: Beilstein J Org Chem. 2026 Mar 17;22:480–5. doi: 10.3762/bjoc.22.35 (PMC13033894; doi:10.3762/bjoc.22.35)
Supplement: File 1 — Chemical protocols and characterization data for compounds, biological protocols including cryo-EM grid prep, data collection, and images of micrographs. [file Beilstein_J_Org_Chem-22-480-s001.pdf]

BEILSTEIN JOURNAL OF ORGANIC CHEMISTRY

Supporting Information

for

Synthesis of a HDAC inhibitor–nanogold probe for cryo-EM visualization in class I HDAC co-repressor complexes

Wiktoria A. Pytel, John W. R. Schwabe and James T. Hodgkinson

Beilstein J. Org. Chem. **2026,** *22,* 480–485. doi:10.3762/bjoc.22.35

Chemical protocols and characterization data for compounds, biological protocols including cryo-EM grid prep, data collection, and images of micrographs

License and Terms: This is a supporting information file under the terms of the Creative Commons Attribution License (https://creativecommons.org/licenses/by/4.0). Please note that the reuse, redistribution and reproduction in particular requires that the author(s) and source are credited and that individual graphics may be subject to special legal provisions.
The license is subject to the *Beilstein Journal of Organic Chemistry* terms and conditions: (https://www.beilstein-journals.org/bjoc/terms)

Table of contents

Supporting figures S2
Supporting methods S6
Chemistry S6
General Information S6
Synthesis of CI-994 ..... S7
Synthesis of the Au–(CI-994) S12
Biology S18
Materials S18
Expression and purification of the HDAC1-CoREST-LSD1 complex S18
Transfection S18
Protein purification S18
SDS-PAGE S20
Protein quantification S21
Histone deacetylase assay S21
Protein cross-linking S22
EM of Au–(CI-994) S22
Cryo-EM grid preparation S22
Cryo-EM grid screening and data collection S23
Cryo-EM data processing S23
References S24

Supporting figures

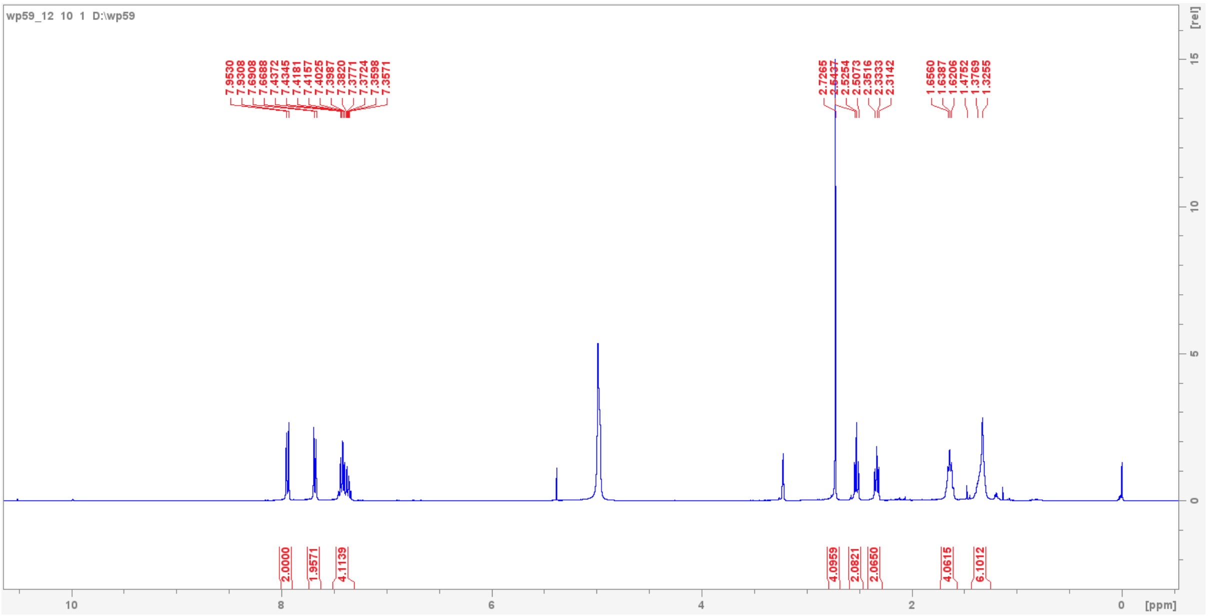

Figure S1 – ^{1}H NMR spectrum of compound **9**.

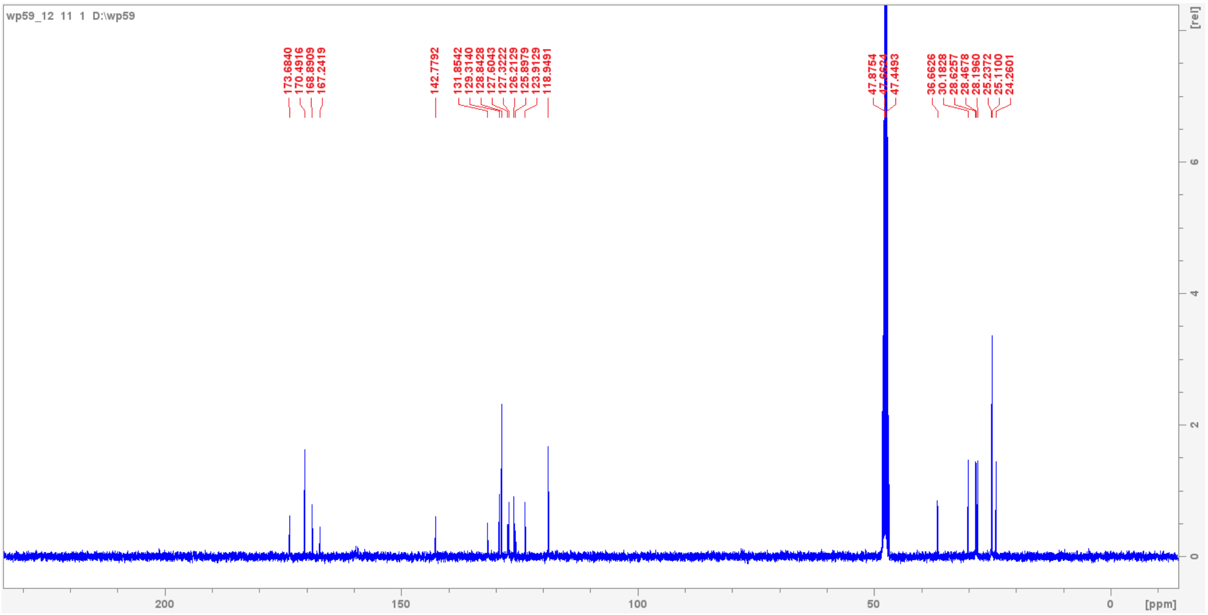

Figure S2 – ^{13}C NMR spectrum of compound **9**.

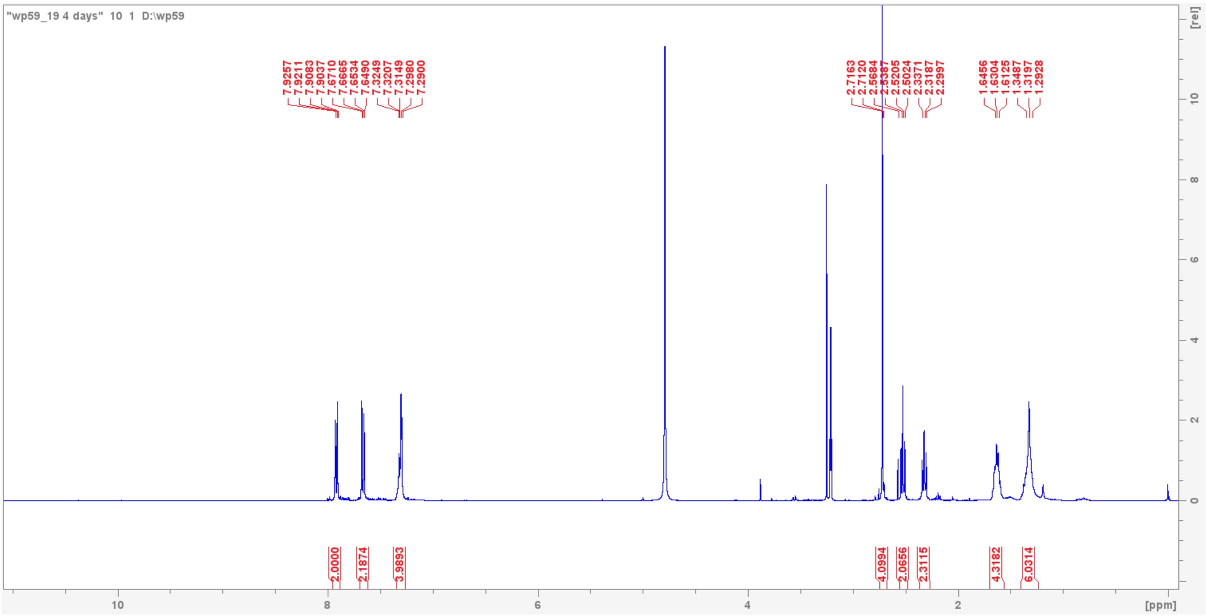

Figure S3 - ^{1}H NMR spectrum of compound **9** after 4 days storage at −20 °C.

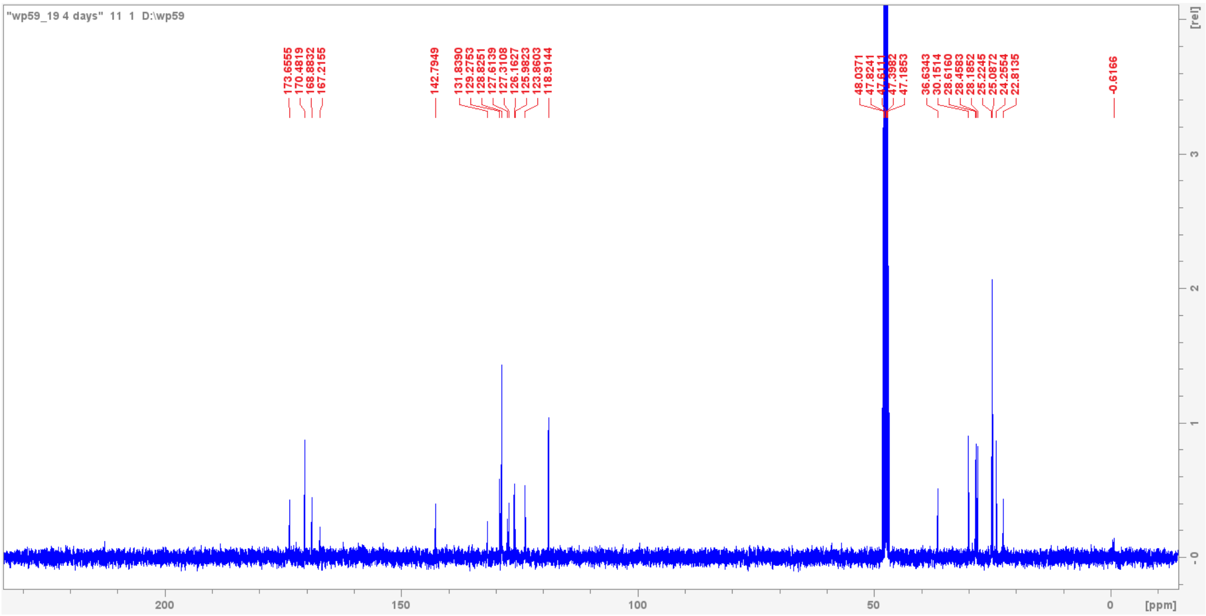

Figure S4 – ^{13}C NMR spectrum of compound **9** after 4 days storage at −20 °C.

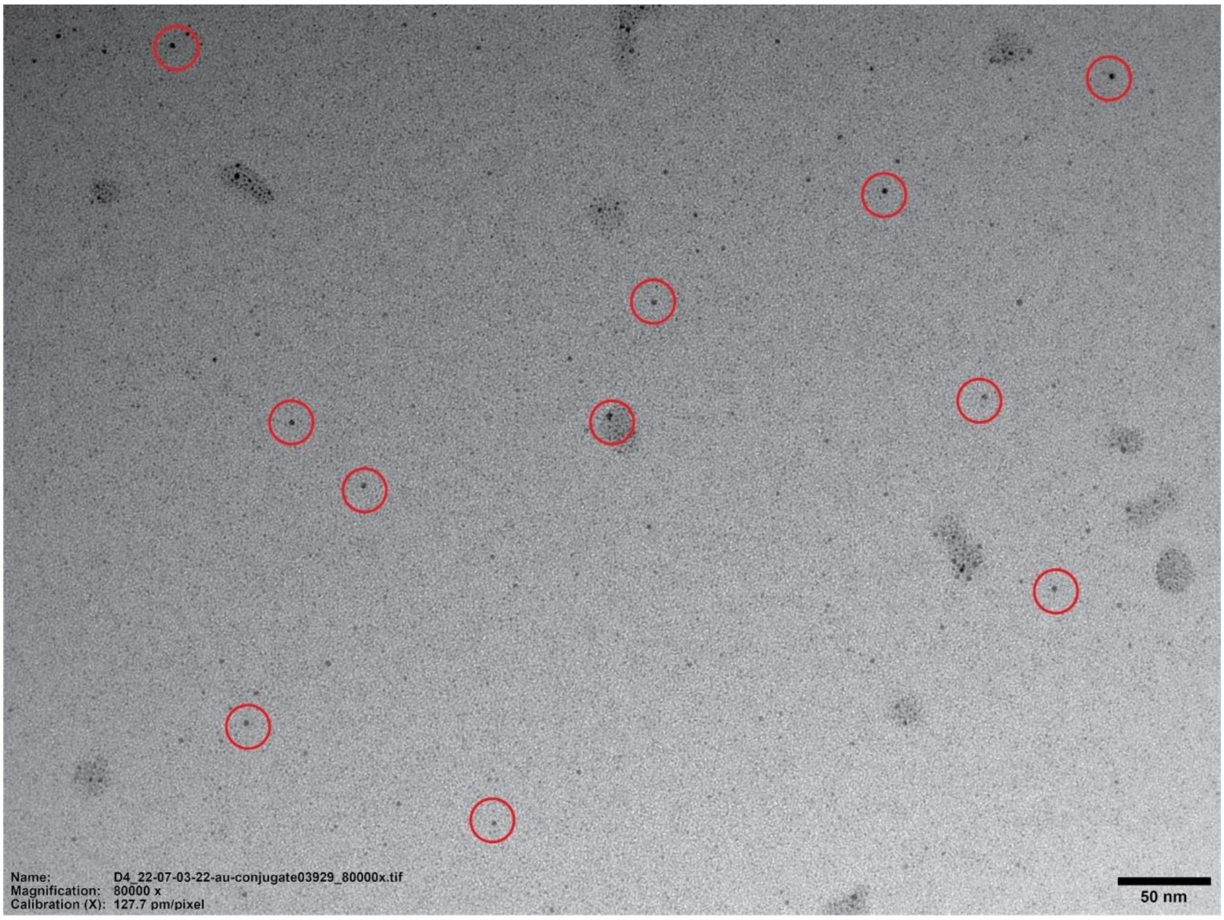

Figure S5 - An EM micrograph of the Au-(CI-994) probe, highlighted in red circles (no stain).

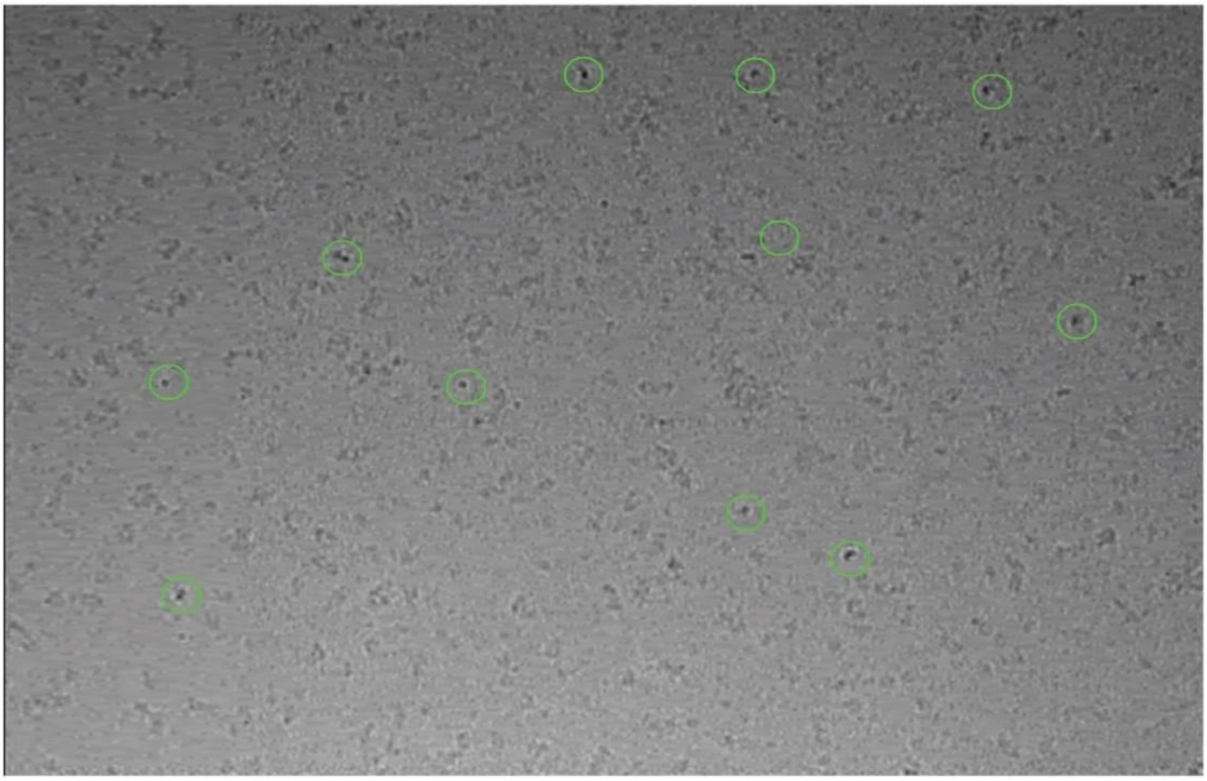

Figure S6 - An example of a cryo-EM micrograph, highlighting particles containing the CoREST ternary complex and the Au-(CI-994) probe (green circles).

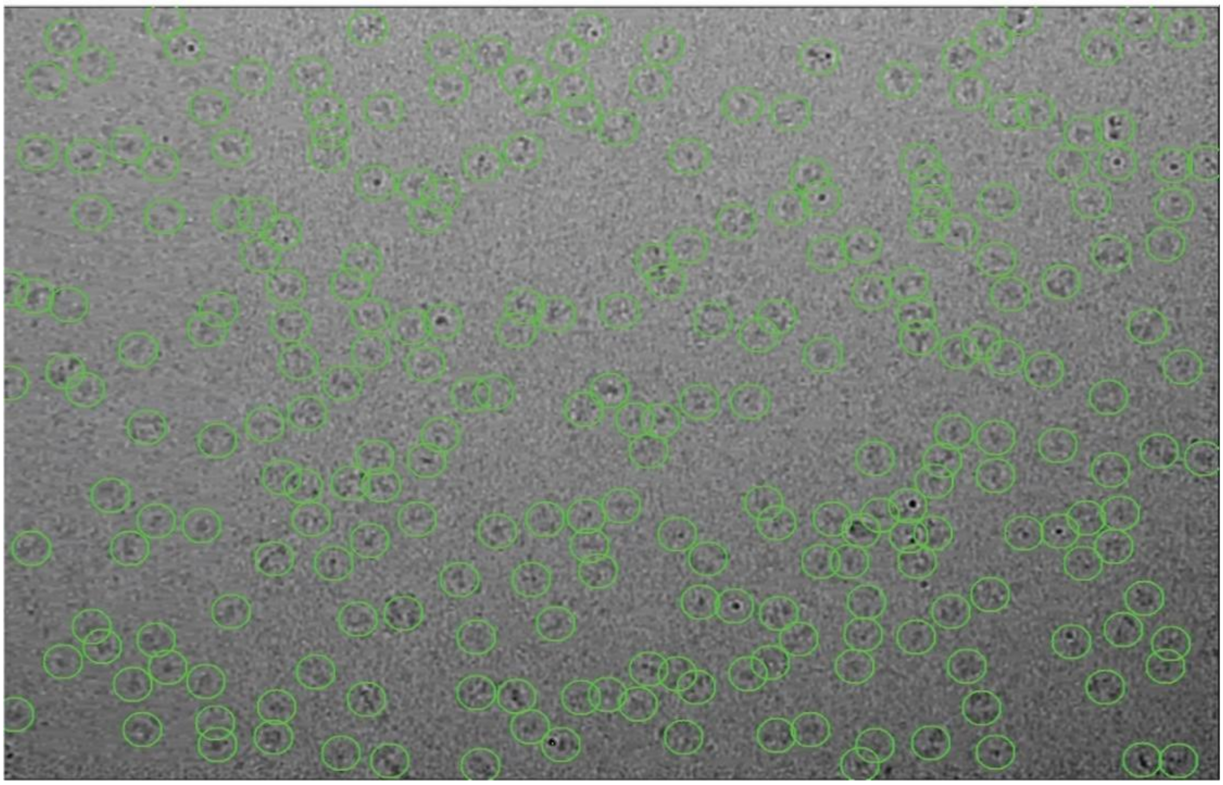

Figure S7 - An example of a cryo-EM micrograph, highlighting particles picked by TOPAZ (green circles).

Supporting methods

Chemistry

General Information

All solvents and reagents were obtained from Fisher Scientific, Sigma Aldrich, Acros Organics and Fluorochem. Macroporous polystyrene-co-divinylbenzene (MP) carbonate resin (3.02 mmol/g loading capacity) was used for neutralizing amine TFA salts and scavenging excess TFA during Boc-deprotection reactions. Monoamino nanogold (1.4 nm) was purchased from Nanoprobes (Product number 2021A-5X6NMOL). For moisture sensitive reaction, the required glassware was dried in an oven at 100 °C for 12 hours and anhydrous solvents were used. Dry DCM and THF were obtained from a Innovative Technology Inc. PureSolv solvent purification system. Unless otherwise stated reactions were performed under nitrogen. Room temperature indicates ambient temperature. Temperatures of 0 °C were maintained using an ice–water bath. Analytical TLCs were run on aluminium-backed silica gel or neutral alumina plates. Purifications were conducted through flash column chromatography with silica gel 60 (230–400 mesh) or neutral activated Brockmann I grade alumina (150 mesh), using commercial solvents. Unless otherwise stated, purifications were performed with silica gel 60. All evaporations in vacuo were carried out under reduced pressure utilizing a Büchi rotary evaporator. Chemical names of synthesized compounds have been generated using ChemDraw Professional.

NMR spectra (^{1}H and ^{13}C) were recorded on a Bruker AV400 machine at ambient temperatures in deuterated solvents ($CDCl_3$, MeOD-d_4 or DMSO-d_6). The solvent used is outlined in the individual compound data. ^{1}H NMR chemical shifts (δ) are given in parts per million (ppm) and reported to the nearest 0.01 ppm with tetramethylsilane used as a reference. Coupling constants (*J*) are calculated in hertz (Hz) to the nearest 0.1 Hz and found using ACD Labs/Spectrus Processor 2019.1.1. ^{13}C NMR spectra

were recorded through broadband proton decoupling. ^{13}C NMR chemical shifts (δ) are given in ppm and are reported to the nearest 0.1 ppm. NMR data are reported in the following order: chemical shift, integration, multiplicity (b, broad; s, singlet; d, doublet; t, triplet; q, quartet; quin, quintet; sept, septet; m, multiplet) or as a combination (dd, doublet of doublets; dt, doublet of triplets;), coupling constant(s) and individual atom assignments. 2D NMR spectroscopy (COSY, HSQC, HMBC) was used to fully assign the ^{1}H NMR and ^{13}C NMR spectra. Mass spectrometry was performed on a Micromass Quatro LC electrospray spectrometer. HRMS was conducted on a Waters Acquity XEVO Q time of flight spectrometer with either electrospray (ES^{+}) or atmospheric solids analysis probe (ASAP) ionization.

Synthesis of CI-994

***tert*-Butyl (2-aminophenyl)carbamate (1)**

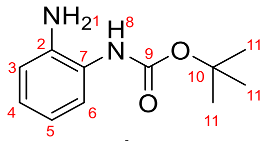

1

Compound **1** was prepared in a manner analogous to [1]. A solution of di-*tert*-butyl dicarbonate (6.11 g, 28.0 mmol) in THF (50 mL) was added dropwise over 3 hours to a solution of *o*-phenylenediamine (3.07 g, 28.4 mmol) and triethylamine (4.6 mL, 33.0 mmol) in THF (50 mL) at 0 °C. The mixture was stirred at room temperature for 19 hours. The reaction mixture was concentrated in vacuo to afford a grey/white/yellow crystalline solid. The solid was redissolved in EtOAc, washed with water (2 × 40 mL) and saturated NaCl (2 × 40 mL), filtered over Na_2SO_4, and concentrated in vacuo to afford a pale yellow/grey solid. The crude solid was purified by flash column chromatography (dry load, 25% EtOAc in hexane) to yield **1** as a white/cream solid

(4.08 g, 13.5 mmol, 70% yield). Spectroscopic data are in accordance with previous literature [1].

^{1}H NMR (400 MHz, $CDCl_3$) δH ppm 7.29 (d, *J* = 8.0 Hz, 1H, H6), 7.01 (td, *J* = 7.7 Hz, 1.5 Hz, 1H, H4), 6.83-6.76 (m, 2H, H3, H5), 6.33 (br s, 1H, H8), 3.76 (br s, 2H, H1), 1.54 (s, 9H, H11).

^{13}C NMR (100 MHz, $CDCl_3$) δC ppm 153.9 (C9), 140.0 (C2), 126.2 (C4, C7), 124.8 (C6), 119.6 (C5), 117.6 (C3), 80.5 (C10), 28.4 (C11).

HRMS (ESI) m/z: $[M+H]^+$ calculated for $C_{11}H_{17}N_2O_2$: 209.1290, found 209.1290.

***tert*-Butyl (2-(4-nitrobenzamido)phenyl)carbamate (2)**

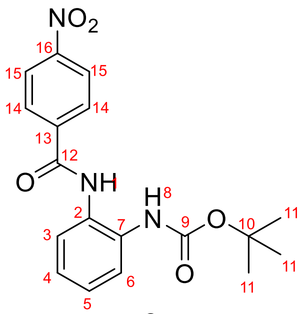

2

Compound **2** was prepared in a manner analogous to [1]. Compound **1** (2.95 g, 14.2 mmol) was dissolved in dry DCM (90 mL) and the solution was cooled to 0 °C. DIPEA (4 mL, 23.0 mmol) was added and the solution was stirred for 20 minutes at 0 °C. Next, 4-nitrobenzoyl chloride (2.93 g, 15.8 mmol) dissolved in DCM (30 mL) was added dropwise to the above solution at 0 °C and stirred for 20 minutes at 0 °C and then at room temperature for 16 hours. The reaction mixture was then diluted with additional DCM (20 mL) and washed with saturated $NaHCO_3$ (100 mL), 1 M HCl (100 mL) and saturated NaCl (100 mL). The organic layer was dried over Na_2SO_4 and concentrated in vacuo to yield a crude yellow solid (5.41 g, 15.1 mmol). The crude product was triturated in EtOH and then filtered to afford **2** as a pale-yellow solid

(3.66 g, 10.2 mmol, 74%). Spectroscopic data are in accordance with previous literature [1].

^{1}H NMR (400 MHz, $CDCl_3$) δH ppm 9.76 (br s, 1H, NH), 8.32 (app d, *J* = 8.9 Hz, 2H, H15), 8.15 (d, *J* = 8.7 Hz, 2H, H14), 7.92 (d, *J* = 8.2 Hz, 1H, H3), 7.29-7.25 (m, 1H, H5), 7.20-7.14 (m, 2H, H4, H6), 6.75 (br s, 1H, NH), 1.53 (s, 9H, H11).

^{13}C NMR (100 MHz, $CDCl_3$) δC ppm 163.2 (C12), 155.0 (C9), 149.8 (C13), 140.0 (C16), 130.7 (C7), 129.4 (C2), 128.6 (C14), 126.4 (C5), 126.3 (C4) 125.9 (C3), 124.4 (C6), 123.8 (C15), 82.0 (C10), 28.3 (C11).

HRMS (ESI) m/z: $[M+Na]^+$ calculated for $C_{18}H_{19}N_3O_5Na$: 380.1222, found 380.1223.

***tert*-Butyl (2-(4-aminobenzamido)phenyl)carbamate (3)**

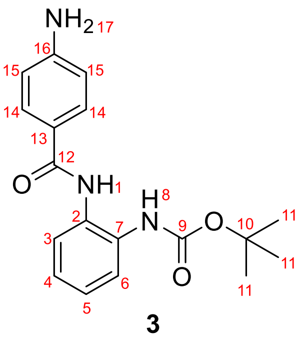

3

Compound **3** was prepared in a manner analogous to [1]. Compound **2** (2.90 g, 8.1 mmol) was dissolved in MeOH/THF 1:1 (80 mL) and 10 wt % Pd/C (0.30 g) was added. The reaction flask was evacuated and filled with nitrogen three times using a Schlenk line. Next, a balloon of hydrogen was added and the reaction mixture was stirred vigorously for 17 hours. The balloon of hydrogen was removed and the flask was evacuated and filled with nitrogen. The reaction mixture was filtered through celite and then the celite was washed further with MeOH (3 × 50 mL). The filtrate was concentrated in vacuo to yield **3** as a white crystalline solid (2.51 g, 7.7 mmol, 95%). Spectroscopic data are in accordance with previous literature [1].

^{1}**H NMR** (400 MHz, $CDCl_3$) δH ppm 8.77 (br s, 1H, NH), 7.79 (app. d, *J* = 8 Hz, 2H, H14), 7.69 (dd, *J* = 7.8 Hz, 1.3 Hz, 1H, H6), 7.30 (dd, *J* = 7.8 Hz, 1.6 Hz, 1H, H3), 7.21-7.12 (m, 2H, H4, H5), 6.86 (br s, 1H, NH), 6.69 (app d, *J* = 8.0 Hz, 2H, H15), 4.02 (br s, 2H, H17), 1.51 (s, 9H, H11).

^{13}**C NMR** (100 MHz, $CDCl_3$) δC ppm 165.5 (C12), 154.5 (C9), 150.0 (C13), 131.2 (C2), 130.1 (C7), 129.3 (C14), 125.9 (C6), 125.7 (C5, C4), 124.5 (C3), 123.7 (C16), 114.1 (C15), 81.1 (C10), 28.3 (C11).

HRMS (ESI) m/z: $[M+Na]^+$ calculated for $C_{18}H_{21}N_3O_3Na$: 350.1481, found 350.1483.

***tert*-Butyl (2-(4-acetamidobenzamido)phenyl)carbamate (4)**

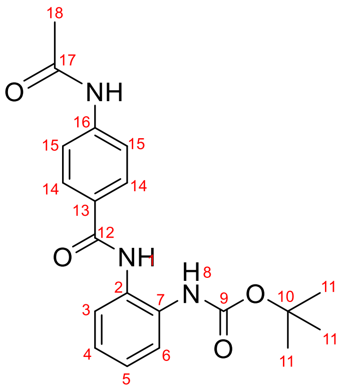

4

Compound **4** was prepared in a manner analogous to [1]. Compound **3** (0.16 g, 0.5 mmol) was dissolved in dry THF (10 mL) and the solution was cooled to 0 °C. NEt_3 (0.2 mL, 1.4 mmol) was then added at 0 °C, followed by a dropwise addition of acetyl chloride (0.05 mL, 0.7 mmol). The resultant mixture was stirred at 0 °C for 15 minutes and then at room temperature for 21 hours. The reaction mixture was then concentrated in vacuo to yield a crude white solid (0.40 g, 1.1 mmol). The crude product was purified by flash column chromatography (dry load, 100 % EtOAc) to afford **4** as a white solid (0.12 g, 0.3 mmol, 67%). Spectroscopic data are in accordance with previous literature [1].

^{1}H NMR (400 MHz, DMSO-d_6) δH ppm 10.26 (s, 1H, NH), 9.76 (s, 1H, NH), 8.70 (br s, 1H, NH), 7.92 (d, *J* = 8.7 Hz, 2H, H14), 7.73 (d, *J* = 8.7 Hz, 2H, H15), 7.55-7.51 (m, 2H, H3, H6), 7.21-7.13 (m, 2H, H4, H5), 2.09 (s, 3H, H18), 1.45 (s, 9H, H11).

^{13}C NMR (100 MHz, DMSO-d_6) δC ppm 169.3 (C17) 165.2 (C12), 154.0 (C9), 143.0 (C16), 132.1 (C2), 130.4 (C7), 129.0 (C14), 128.7 (C13), 126.5 (C3/6), 126.0 (C4/5), 124.6 (C3/6), 124.4 (C4/5), 118.6 (C15), 80.1 (C10), 28.5 (C11), 24.6 (C18).

HRMS (ESI) m/z: $[M+H]^+$ calculated for $C_{20}H_{24}N_3O_4$: 370.1767, found 370.1765.

4-Acetamido-*N*-(2-aminophenyl)benzamide (CI-994)

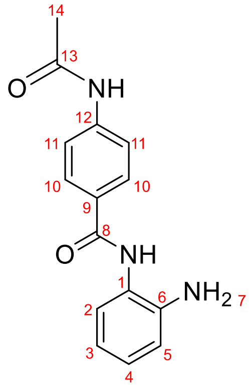

CI-994

CI-994 was prepared in a manner analogous to [1]. Compound **4** (0.08 g, 0.2 mmol) was dissolved in dry DCM (15 mL) and the solution was cooled to 0 °C. Next, TFA (0.2 mL, 2.6 mmol) was added dropwise at 0 °C and the reaction mixture was stirred for 20 hours. After an overnight stir, the reaction mixture was concentrated in vacuo to afford a brown oil with a white precipitate (0.13 g, 0.5 mmol). The compound was re-dissolved in MeOH (25 mL) and agitated in MP-carbonate resin (3.02 mmol/g loading capacity, 0.35 g) for 3 hours. The mixture was then filtered and the filtrate was concentrated in vacuo to yield crude CI-994 as a pale yellow/white solid (0.07 g, 0.2 mmol). The crude product was purified on column chromatography (dry load, silica,

1% MeOH in EtOAc) to afford pure CI-994 as a white solid (0.06 g, 0.2 mmol, 95%). Spectroscopic data are in accordance with previous literature [1].

^{1}H NMR (400 MHz, DMSO-d$_6$) δH ppm 10.19 (s, 1H, NH), 9.56 (s, 1H, NH), 7.94 (d, *J* = 8.7 Hz, 2H, H10), 7.69 (d, *J* = 8.7 Hz, 2H, H11), 7.16 (d, *J* = 7.7 Hz, 1H, H2), 6.96 (t, *J* = 7.7 Hz, 1H, H4), 6.78 (dd, *J* = 8.0 Hz, 1.3 Hz, 1H, H5), 6.59 (t, *J* = 7.6 Hz, 1H, H3), 4.87 (s, 2H, H7), 2.09 (s, 3H, H14).

^{13}C NMR (100 MHz, DMSO-d$_6$) δC ppm 169.2 (C13) 165.2 (C8), 143.6 (C6), 142.6 (C12), 129.2 (C9), 129.1 (C10), 127.1 (C2), 126.8 (C4), 124.0 (C1), 118.5 (C11), 116.7 (C3), 116.6 (C5), 24.6 (C14).

HRMS (ESI) m/z: $[M+Na]^+$ calculated for $C_{15}H_{15}N_3O_2Na$: 292.1062, found 292.1061.

Synthesis of Au–(CI-994)

9-(Benzyloxy)-9-oxononanoic acid (5)

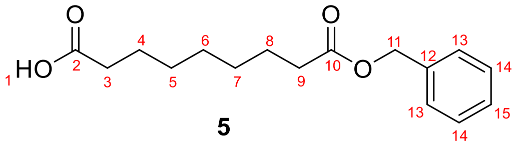

5

Compound **5** was prepared in a manner analogous to [2]. Nonanedioic acid (2.54 g, 13.5 mmol) was dissolved in 1,4 dioxane/DMF solution 1:1 (50 mL). Benzyl bromide (1.6 mL, 13.5 mmol) was added, followed by $NaHCO_3$ (1.25 g, 15.0 mmol). The resulting suspension was heated at 90 °C for 19 hours. The reaction mixture was allowed to cool and was concentrated in vacuo*.* The cloudy suspension was then suspended in EtOAc (60 mL) and washed with saturated NaCl solution (60 mL) and water (60 mL). The organic phase was collected, dried over $MgSO_4$, filtered and concentrated in vacuo to afford crude **5** as a creamy oil (3.59 g, 12.9 mmol). The crude product was purified using flash column chromatography (50% EtOAc in hexane) to

yield **5** as a pale-yellow oil (1.68 g, 6.0 mmol, 45%). Spectroscopic data are in accordance with previous literature [2].

^{1}H NMR (400 MHz, $CDCl_3$) δH ppm 10.42 (br s, 1H, H1), 7.36-7.34 (m, 5H, H13, H14, H15), 5.11 (s, 2H, H11), 2.37-2.31 (m, 4H, H3, H9), 1.65-1.58 (m, 4H, H4, H8), 1.37-1.26 (m, 6H, H5, H6, H7).

^{13}C NMR (100 MHz, $CDCl_3$) δC ppm 179.9 (C2), 173.7 (C10), 136.1 (C12), 128.5 (C14), 128.19 (C13), 128.18 (C15) 66.1 (C11), 34.3 (C9), 34.0 (C3), 28.9 (C5/6/7), 28.8 (2 x CH_2, C5/6/7), 24.9 (C8), 24.6 (C4).

HRMS (ESI) m/z: $[M+Na]^+$ calculated for $C_{16}H_{22}O_4Na$: 301.1416, found 301.1419.

Benzyl 9-((4-((2-((tert-butoxycarbonyl)amino)phenyl)carbamoyl)phenyl)amino)-9 oxononanoate (6)

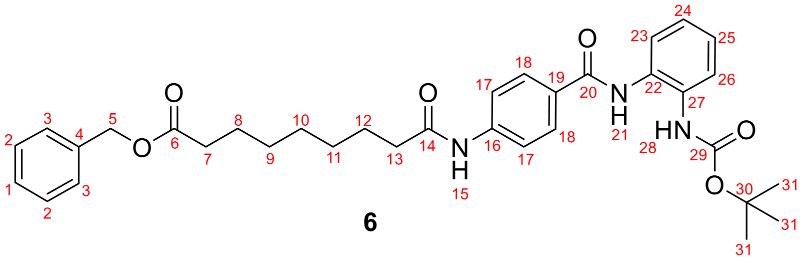

6

Compound **6** was prepared in a manner analogous to [2]. A solution of compound **5** (0.95 g, 3.4 mmol) in dry DMF (30 mL) was cooled to 0 °C and HATU (1.5 g, 3.9 mmol) and DIPEA (1.8 mL, 10.3 mmol) were added. The reaction mixture was stirred at 0 °C for 30 minutes and then compound **3** (0.86 g, 2.6 mmol) in dry DMF (5 mL) was added. The resultant mixture was stirred at room temperature for 40 hours. The reaction mixture was then diluted with EtOAc (30 mL) and washed with saturated $NaHCO_3$ (2 × 20 mL) and saturated NaCl solution (2 × 20 mL). The organic layer was dried over $MgSO_4$, filtered and the solvent was evaporated in vacuo to yield crude **6** as a brown oil (2.03 g, 3.5 mmol). The crude product was purified using flash column

chromatography (20–50% EtOAc in hexane) to afford **6** as a yellow gum (0.9 g, 1.5 mmol, 59 %). Spectroscopic data are in accordance with previous literature [2].

^{1}H NMR (400 MHz, $CDCl_3$) δH ppm 9.39 (br s, 1H, NH), 8.56 (br s, 1H, NH), 7.83 (d, *J* = 8.7 Hz, 2H, H18), 7.62-7.60 (m, 1H, H23), 7.56 (d, *J* = 8.6 Hz, 2H, H17) 7.42 (s, 1H, NH), 7.34-7.29 (m, 6H, H1, H2, H3, H26), 7.10-7.05 (m, 2H, H24, H25), 5.10 (s, 2H, H5), 2.33 (t, *J* = 7.5 Hz, 2H, H7), 2.27 (t, *J* = 7.5 Hz, 2H, H13), 1.66-1.56 (m, 4H, H8, H12), 1.46 (s, 9H, H31), 1.30-1.22 (m, 6H, H9, H10, H11).

^{13}C NMR (100 MHz, $CDCl_3$) δC ppm 174.9 (C6), 172.4 (C14), 165.7 (C20), 154.7 (C29), 141.9 (C16), 136.0 (C4), 130.8 (C22), 130.4 (C27), 128.8 (C19), 128.6 (C2), 128.5 (C18), 128.3 (C1), 128.1 (C3), 126.1 (C24), 125.9 (C23), 125.4 (C25), 124.4 (C26), 119.2 (C17), 81.2 (C30), 66.2 (C5), 37.4 (C13), 34.3 (C7), 29.0 (2 x CH_2, C9/10/11), 28.9 (C9/10/11), 28.3 (C31), 25.4 (C12), 24.8 (C8).

HRMS (ESI) m/z: $[M+Na]^+$ calculated for $C_{36}H_{41}N_3O_6Na$: 610.2893, found 610.2898.

9-((4-((2-((tert-Butoxycarbonyl)amino)phenyl)carbamoyl)phenyl)amino)-9-oxononanoic acid (7)

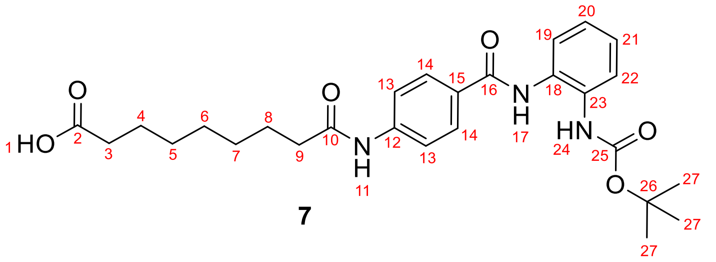

7

Compound **7** was prepared in a manner analogous to [2]. Compound **6** (0.73 g, 1.2 mmol) was dissolved in THF (40 mL) and 10% Pd/C was added (0.08 g). The reaction flask was evacuated and filled with nitrogen three times using a Schlenk line. Next, a balloon of hydrogen was added and the reaction mixture was stirred vigorously for 19 hours. The balloon of hydrogen was removed and the flask was evacuated and filled with nitrogen. The reaction mixture was filtered through celite and then the celite

was washed further with THF (3 × 25 mL). The filtrate was concentrated in vacuo to yield **7** as a white crystalline solid (0.62 g, 1.3 mmol). Spectroscopic data are in accordance with previous literature [2].

^{1}H NMR (400 MHz, DMSO-d$_6$) δH ppm 11.97 (br s, 1H, H1), 10.17 (s, 1H, NH), 9.75 (s, 1H, NH), 8.68 (br s, 1H, NH), 7.92 (d, *J* = 8.8 Hz, 2H, H14), 7.75 (d, *J* = 8.8 Hz, 2H, H13), 7.54 (m, 2H, H19, H22), 7.21-7.13 (m, 2H, H20, H21), 2.35 (t, *J* = 7.4 Hz, 2H, H9), 2.20 (t, *J* = 7.3 Hz, 2H, H3), 1.61 (m, 2H, H8), 1.52-1.48 (m, 2H, H4), 1.45 (s, 9H, H27), 1.33-1.25 (m, 6H, H5, H6, H7).

^{13}C NMR (100 MHz, DMSO-d$_6$) δC ppm 175.0 (C2), 172.3 (C10), 165.2 (C16), 154.0 (C25), 143.0 (C12), 132.1 (C23), 130.4 (C18), 129.0 (C14), 128.0 (C15), 126.4 (C19), 125.9 (C21), 124.6 (C20), 124.4 (C22), 118.7 (C13), 80.1 (C26), 36.9 (C9), 34.1 (C3), 29.0 (2 x CH$_2$, C5/6/7), 28.9 (C5/6/7), 28.5 (C27), 25.4 (C8), 25.0 (C4).

HRMS (ESI) m/z: [M+Na]$^+$ calculated for $C_{27}H_{35}N_3O_6Na$: 520.2424, found 520.2440.

2,5-Dioxopyrrolidin-1-yl 9-((4-((2-((*tert*-butoxycarbonyl)amino)phenyl)carbamoyl)phenyl)amino)-9-oxononanoate (8)

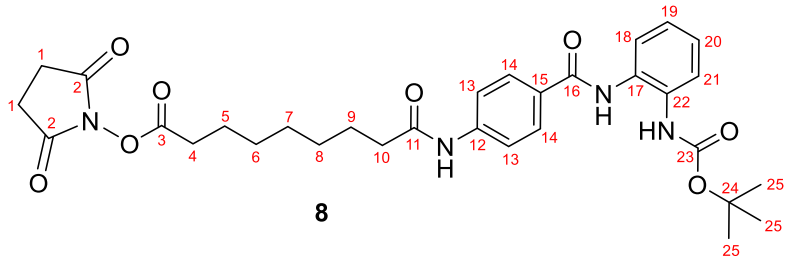

8

To a solution of **7** (0.09 g, 0.2 mmol) in dry DMF (20 mL) at 0 °C NHS (0.07 g, 0.6 mmol) and EDCI (0.05 g, 0.3 mmol) were added. The reaction mixture was stirred at room temperature for 16 hours. Next, the reaction mixture was concentrated in vacuo and the resulting creamy orange oil was redissolved in DCM (40 mL). The organic layer was washed with water (2 × 25 mL) and saturated NaCl solution (3 × 25 mL). The organic layers were dried over anhydrous Na_2SO_4, filtered and concentrated in vacuo to afford crude **8** as an orange oil (0.11 g, 0.2 mmol). The crude product was purified

using flash column chromatography (100% EtOAc) to afford **8** as a pale orange oil (0.09 g, 0.2 mmol, 90%).

^{1}H NMR (400 MHz, $CDCl_3$) δH ppm 9.20 (br s, 1H, NH), 8.02 (br s, 1H, NH), 7.88 (d, *J* = 8.0 Hz, 2H, H14), 7.70 (d, *J* = 7.6 Hz, 1H, H18), 7.61 (d, *J* = 8.1 Hz, 2H, H13), 7.29 (d, *J* = 7.5 Hz, 1H, H21), 7.19-7.11 (m, 2H, H19, H20), 7.03 (s, 1H, NH), 2.82 (s, 4H, H1), 2.58 (t, *J* = 7.2 Hz, 2H, H10), 2.33 (t, *J* = 7.5 Hz, 2H, H4), 1.76-1.66 (m, 4H, H5, H9), 1.50 (s, 9H, H25), 1.44-1.30 (m, 6H, H6, H7, H8).

^{13}C NMR (100 MHz, $CDCl_3$) δC ppm 172.0 (C11), 169.4 (C2), 168.7 (C3), 165.2 (C16), 154.6 (C23), 141.6 (C12), 130.7 (C22), 130.3 (C17), 129.2 (C15), 128.5 (C14), 126.0 (C20), 125.8 (C18), 125.6 (C19), 124.5 (C21), 119.1 (C13), 81.3 (C24), 37.5 (C4), 30.9 (C10), 28.7 (C6/7/8), 28.4 (2 x CH_2, C6/7/8), 28.3 (C25), 25.6 (C1), 25.1 (C9), 24.4 (C5).

HRMS (ESI) m/z: $[M+Na]^+$ calculated for $C_{31}H_{38}N_4O_8Na$: 617.2588, found 617.2584.

2-(4-(9-((2,5-Dioxopyrrolidin-1-yl)oxy)-9-oxononanamido)benzamido)benzene-aminium trifluroacetate (9)

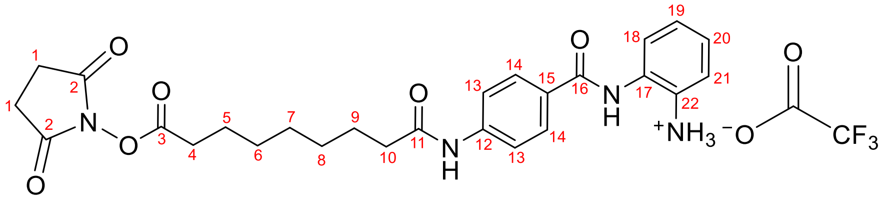

9

Compound **8** (0.11 g, 0.2 mmol) was dissolved in dry DCM (10 mL) and the reaction flask was evacuated and filled with nitrogen using a Schlenk line two times. The solution was cooled to 0 °C and then TFA (0.6 mL, 3.6 mmol) was added. The resulting reaction mixture was stirred at room temperature for 18 hours. The reaction mixture was then concentrated in vacuo to afford **9** as a brown oil (0.10 g, 0.2 mmol, 92%).

^{1}H NMR (400 MHz, MeOD-d$_4$) δH ppm) 7.94 (d, *J* = 8.9 Hz, 2H, H14), 7.68 (d, *J* = 8.8 Hz, 2H, H13), 7.46-7.34 (m, 4H, H18, H19, H20, H21), 2.73 (s, 4H, H1), 2.53 (t, *J* = 7.3 Hz, 2H, H10), 2.33 (t, *J* = 7.5 Hz, 2H, H4), 1.69-1.58 (m, 4H, H9, H5), 1.40-1.29 (m, 6H, H6, H7, H8).

^{13}C NMR (100 MHz, MeOD-d$_4$) δC ppm 173.7 (C11), 170.5 (C2), 168.9 (C3), 167.2 (C16), 142.8 (C12), 131.9 (C15), 129.3 (C19), 128.8 (C14), 127.6 (C17) 127.3 (C20), 126.2 (C18), 125.9 (C22), 123.9 (C21), 119.0 (C13), 36.7 (C4), 30.2 (C10), 28.6 (C6/7/8), 28.5 (C6/7/8), 28.2 (C6/7/8), 25.2 (C9), 25.1 (C1), 24.3 (C5).

HRMS (ESI) m/z: $[M+H]^+$ calculated for $C_{26}H_{31}N_4O_6$: 495.2238, found 495.2241.

Note: ^{19}F NMR confirmed the presence of TFA counterion.

Au–(CI-994)

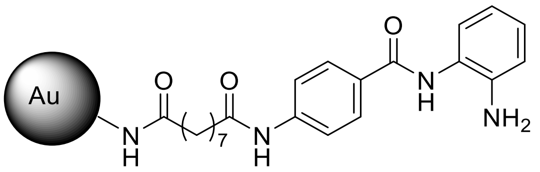

Au-(CI-994)

Compound **9** (83 µL, 8.2 mM) dissolved in dry DMSO was added to Au–NH$_2$ (Nanoprobes, Monoamino nanogold 1.4 nm, 2021A-5X6NMOL, 417 µL, 12 µM), dissolved in dry DMSO and the reaction mixture was stirred vigorously under N_2 for 20 hours. After 20 hours, the reaction mixture was diluted with ddH$_2$O (5 mL) and transferred to an Amricon concentrator, with a 10 kDa molecular weight cut-off. The reaction mixture was concentrated to smaller volumes (2000 rpm, 10 minutes) and then further washed with ddH$_2$O (15 mL). Following the ddH$_2$O wash, the Au–(CI-994) was concentrated to 1 mL. Concentration of Au–(CI-994) (2.69 µM) was determined by Abs$_{420nm}$, based on Beer–Lambert law (A = 0.042, ε = 1.56 × 10^5 M^{-1} cm^{-1}, l = 0.1 cm).

Biology

Materials

Unless otherwise stated, chemicals and reagents were of analytical grade or higher and purchased from Sigma Aldrich, Fisher Scientific, MELFORD and Merck Millipore. Mammalian cell culture medium, FreeStyle™293 expression medium, was purchased from Gibco® Life technologies™

Expression and purification of the HDAC1-CoREST-LSD1 complex

Transfection

HEK293F cells were used to express the ternary HDAC1-CoREST-LSD1 complex. For a 300 mL of cells transfection, 300 µg of total DNA (in 1:1:1 construct ratios) was diluted in 30 mL of sterile PBS (Sigma). 600 µL of the polyethyleneimine (PEI) transfection reagent (1 mg/mL) was added to the mixture. The mixture was shaken vigorously and then incubated at room temperature for 30 minutes. The volumes and quantities of reagents were scaled proportionally to the working cell volumes. The PBS:DNA:PEI mixture was added to the cells at the density of 1×10^6 cells per mL. The cells were incubated in a 5% CO_2 shaking incubator (N-BIOTEK) at 120 rpf, 37 °C and then harvested by spinning at 4000 rpm (3501*g*, Sorvall SLC-6000) for 15 minutes at 4 °C after a 48-hour incubation. Harvested cells were either directly used for purification or stored at −80 °C.

Protein purification

The cell pellet from a 1.2 L transfection was dissolved in 30 mL of lysis buffer and then lysed by sonication. The sonicator (QSONICA) was set up to perform 4 cycles of 15 seconds on, 15 seconds off sonication with an amplitude of 40%. The insoluble material was removed by spinning at 30,000 rpm for 20 minutes at 4 °C (108,000*g*, Avanti™ J-30I, Beckman Coulter, Rotor JA30.50). The supernatant was transferred to a falcon tube containing 1 mL of anti-FLAG M2 resin (Sigma). The resin was pre-washed with 10 mL of lysis buffer. The protein–resin mixture was incubated for

30 minutes at 4 °C on a tube roller. After incubation, the protein–resin mixture was washed once with 10 mL of lysis buffer, four times with 10 mL of wash buffer and four times with 10 mL of cleavage buffer. After the washing steps, the protein–resin mixture was resuspended in 4 mL of cleavage buffer. Then, 30–40 µL of 1.2 mg/mL Tev protease was added to the mixture and it was incubated overnight at 4 °C on a tube roller. A 10 µL sample was taken before addition of Tev protease as a control sample for SDS-PAGE. After the Tev cleavage, the protein was concentrated to 500 µL using a 4 mL Amicon®Ultra centrifugal filter (Merck Millipore) with a membrane nominal molecular weight cut off of 10 kDa. The concentrated protein was then filtered through a 0.22 µm centrifugal filter (Merck Millipore). The protein complex was purified on the AKTA purifier system (GE Healthcare) using a Superose 6 (10/300 GL) column (GE Healthcare), which was equilibrated in the gel filtration buffer before sample loading. Fractions were collected in a 96-well plate (0.5 mL per fraction). A 10 µL sample from the fraction, which showed absorbance at 280 nm, was taken for analysis by SDS-PAGE. Fractions containing purified protein were concentrated using a 0.5 mL Amicon Ultra centrifugal filter (Merck Millipore) with a membrane nominal molecular weight cut off of 10 kDa.

Table S1 – Composition of buffers used in protein purification.

Lysis buffer	Wash buffer	Cleavage buffer	Gel filtration buffer (standard)	Gel filtration buffer (EM)
50 mM Tris/Cl pH 7.5	50 mM Tris/Cl pH 7.5	50 mM Tris/Cl pH 7.5	25 mM Tris/Cl pH 7.5	25 mM HEPES pH 7.5
50 mM KOAc	50 mM KOAc	50 mM KOAc	50 mM KOAc	50 mM KCl
5% v/v Glycerol	5% v/v glycerol	5% v/v glycerol	0.5 mM TCEP	
0.4% v/v Triton X-100		0.5 mM TCEP		
Roche complete EDTA free protease inhibitor (1 tablet per 50 mL)				

SDS-PAGE

2 µL of the 6 × SDS sample buffer (60% glycerol, 375 mM Tris/Cl pH 6.8, 12% SDS, 300 mM DTT and 0.3 mg/mL bromophenol blue) was added to 10 µL of protein sample. Samples were run on a NuPAGE® 4–12% Bis-Tris gel (Novex) with NuPAGE® MES SDS running buffer at 200 V/125 mA/100 W until the bromophenol blue dye was at the

bottom of gel. SeeBlue Plus2 prestained marker (Invitrogen) was used as the molecular weight marker and the gel was stained using Instant Blue Coomassie Stain (Expedeon).

Protein quantification

Concentration of the protein was determined using a protein assay dye reagent (Bio-Rad). 200 μL of the reagent was diluted in 800 μL of ddH_2O and used as a reagent blank. Then, 2 μL of protein sample was added into this measurement mixture and the absorbance was measured at 595 nm. The concentration of the protein sample in mg/mL was then calculated by multiplying by '17', which represents a factor calculated from a BSA standard curve.

Histone deacetylase assay

HDAC activity and inhibition experiments against the ternary CoREST complex were carried out using a fluorescent HDAC assay in a manner analogous to [1]. The assays were conducted on black 96-well plates (Corning #3915) with a final reaction volume of 50 μL. 100 μM of Boc-(Ac)-Lys-AMC was used as a substrate in each well. All determinations were performed in triplicate. CI-994 was dissolved at 50 mM in DMSO, and then further diluted with 10% DMSO HDAC assay buffer (10% DMSO, 50 mM Tris pH 7.5, 50 mM NaCl, 0.1 mg/mL BSA) to micromolar concentrations. Au–(CI-994) was concentrated to 2.69 μM and used in the assay at this concentration. Serial dilutions of the inhibitor/probe were then carried out using 10% DMSO HDAC assay buffer to afford a range of concentrations. 10 μL of each of these solutions were added to individual wells, followed by addition of 30 μL of the CoREST complex (12.5 nM) dissolved in HDAC assay buffer (50 mM Tris pH 7.5, 50 mM NaCl, 0.1 mg/mL BSA). The HDAC complex and compound mixture was then incubated at 20 °C, 100 rpm for 2 hours. After incubation, 10 μL of the Boc-(Ac)Lys-AMC substrate, dissolved in HDAC assay buffer, was added to each well. The plate was incubated at 30 °C, 100 rpm for

1 hour, followed by addition of 50 μL of a developer buffer (50 mM Tris pH 7.5, 100 mM NaCl, 10 mg/mL trypsin) to quench the reaction. The reaction was allowed to develop for 10 minutes at 30 °C, 100 rmp. Fluorescence intensity was determined with a Victor X5 plate reader (Perkin Elmer, λ_{ex} = 335 nm, λ_{em} = 460 nm). IC_{50} values were calculated using the GraphPad Prism 7 software by non-linear regression, log(inhibitor) vs. response − variable slope (four parameters).

Protein cross-linking

The CoREST ternary complex was purified as described above and gel filtrated in 25 mM HEPES pH 7.5 and 50 mM KCl. The peak CoREST gel filtration fraction was then concentrated to 0.8 mg/mL and mixed with 0.25% glutaraldehyde in a v/v ratio of 1:1. The reaction was then incubated on ice for 20 minutes before being stopped with Tris/Cl pH 7.5 to a final concentration of 50 mM.

EM of Au–(CI-994)

Formvar/Carbon coat grids (S162 Formvar/Carbon 200-mesh, copper, Elektron technologies) were placed on a glass slide and glow-discharged in an auto sputter coater (E5200, Quorum Technologies). Then, 5 μL of Au–(CI-994) was applied onto a grid and incubated for 1 min. The excess sample was then blotted with filter paper. The grids were left to air-dry for 5 minutes, before being screened on a JEOL JEM-1400 transmission electron microscope.

Cryo-EM grid preparation

Quantifoil grids (300-mesh, R1.2/1.3, gold with holey carbon) were glow-discharged at 35 mA/60 seconds (GloQube, Quorum Technologies). Sample addition, blotting and freeze plunging was performed by the Vitrobot Mark IV (Thermo Fisher Scientific), with the chamber set to 100% humidity and a temperature of 4 °C. A 3 μL sample was applied onto the grid, with a blotting time of 3 seconds and a blot force of 10. Grids were then plunged in liquid ethane and stored in a liquid nitrogen storage dewar.

Cryo-EM grid screening and data collection

Grids were clipped into cartridges, inserted into a Krios nanocap and loaded into the Titan Krios G3 (Thermo Fisher). Grids were screened using low dose mode to check for protein distribution/aggregation and ice thickness. Data was collected on grids with good particle distribution and ice thickness. The microscope was operated at 300 kV with a Gatan Quantum Energy filter camera and a Volta Phase Plate, with a defocus range of −1.3 to −2.8 in 0.3 μm intervals. A Falcon 3 camera was utilized with a nominal magnification of 105000 × and a calibrated physical pixel size of 0.835 Å. Movies were collected over 50 fractions with a detector dose rate of 15 electrons per pixel per second in counting mode over 60 s.

Cryo-EM data processing

A Graphics Processing Unit (GPU) cluster facilitated by Dr TJ Ragan (Leicester Institute of Structural and Chemical Biology) was used to process the data. Data processing was performed using RELION3 or RELION4 [3,4]. The movies collected were motion-corrected using Zheng's MOTIONCOR2 [5]. The contract transfer function (CTF) of the micrographs was corrected using GCTF [6]. Approximately 100 protein particles containing nanogold were manually picked to generate a template for the TOPAZ autopicker [7]. After the particles were picked by TOPAZ, several rounds of 2D classification of particles were performed. Due to resolution of the model, data processing was not continued further.

References

1. Smalley, J.P.; Adams, G.E.; Millard, C.J.; Song, Y.; Norris, J.K.S.; Schwabe, J.W.R.; Cowley, S.M.; Hodgkinson, J.T., *Chem. Commun.,* **2020**, *56*, 4476-4479.

2. Smalley, J.P.; Baker, I.M.; Pytel, W.A.; Lin, L.Y.; Bowman, K.J.; Schwabe, J.W.R.; Cowley, S.M; Hodgkinson, J.T., *J. Med. Chem.,* **2022**, *65*, 5642-5659.

3. Zivanov, J.; Nakane, T.; Forsberg, B. O.; Kimanius, D.; Hagen, W. J.H.; Lindahl E., Scheres, S. H., *eLife*, **2018**, *7*, e42166.

4. Zivanov, J.; Otón, J.; Ke, Z.; Kügelgen, A. von; Pyle, E.; Qu, K.; Morado, D.; Castaño-Díez, D.; Zanetti, G.; Bharat, T. A.M.; Briggs J. A.G., Scheres, S.H.W., *eLife*, **2022**, *11*, e83724.

5. Zheng, S. Q.; Palovcak, E.; Armache, J.-P.; Verba, K. A.; Cheng Y.; Agard, D. A., *Nat Methods*, **2017**, *14*, 331–332.

6. Zhang, K., *J Struct Biol*, **2016**, *193*, 1–12.

7. Bepler, T.; Morin, A.; Rapp, M.; Brasch, J.; Shapiro, L.; Noble A.J.; Berger, B., *Nat Methods*, **2019**, *16*, 1153–1160.
